# Extracorporeal membrane oxygenation in acute respiratory distress syndrome due to influenza A (H1N1)pdm09 pneumonia. A single-center experience during the 2013-2014 season

**DOI:** 10.5935/0103-507X.20170048

**Published:** 2017

**Authors:** Nithya Menon, Carlos M. Perez-Velez, Jennifer A. Wheeler, Michael F. Morris, Orazio L. Amabile, Mark R. Tasset, Robert A. Raschke

**Affiliations:** 1 Division of Pulmonary and Critical Care Medicine, Department of Medicine, Banner - University Medical Center Phoenix - Arizona, United States.; 2 Division of Infectious Diseases, Department of Medicine, Banner - University Medical Center Phoenix - Arizona, United States.; 3 Division of Cardiothoracic Surgery, Department of Surgery, Banner - University Medical Center Phoenix - Arizona, United States.; 4 Division of Thoracic Radiology, Department of Radiology, Banner - University Medical Center Phoenix - Arizona, United States.

**Keywords:** Extracorporeal membrane oxygenation, Respiratory distress syndrome, acute, Influenza A virus, H1N1 subtype

## Abstract

**Objective:**

This report aimed to describe the outcomes of the patients with severe H1N1 associated acute respiratory distress syndrome who were treated with extracorporeal membrane oxygenation therapy.

**Methods:**

This retrospective review analyzed a single-center cohort of adult patients with H1N1-related acute respiratory distress syndrome who were managed with veno-venous extracorporeal membrane oxygenation during the winter of 2013/2014.

**Results:**

A total of 10 patients received veno-venous extracorporeal membrane oxygenation for H1N1 influenza between January 2013 and March 2014. Seven patients were transferred to our center for extracorporeal membrane oxygenation consideration (all within 72 hours of initiating mechanical ventilation). The median patient age was forty years, and 30% were female. The median arterial oxygen partial pressure to fraction of inspired oxygen ratio was 62.5, and the median RESP score was 6. Three patients received inhaled nitric oxide, and four patients were proned as rescue therapy before extracorporeal membrane oxygenation was initiated. The median duration of mechanical ventilation was twenty-two days (range, 14 - 32). The median length of stay in the intensive care unit was twenty-seven days (range, 14 - 39). The median hospital length of stay was 29.1 days (range, 16.0 - 46.9). Minor bleeding complications occurred in 6 of 10 patients. Eight of the ten patients survived to hospital discharge.

**Conclusion:**

The survivors were relatively young and discharged with good functional status (i.e., enhancing quality-adjusted life-years-saved). Our experience shows that even a relatively new extracorporeal membrane oxygenation program can play an important role in that capacity and provide excellent outcomes for the sickest patients.

## Abstract (pt)

**Objetivo:**

Descrever os desfechos de pacientes com síndrome do desconforto respiratório agudo associada à influenza subtipo H1N1 grave tratados com oxigenação por membrana extracorpórea.

**Métodos:**

Trata-se de revisão retrospectiva de uma coorte de pacientes oriunda de um único centro, constituída por adultos com síndrome do desconforto respiratório agudo relacionada com influenza subtipo H1N1 e tratados com oxigenação venovenosa por membrana extracorpórea durante a temporada de inverno no hemisfério norte de 2013/2014.

**Resultados:**

Dez pacientes receberam oxigenação venovenosa por membrana extracorpórea para tratamento de influenza subtipo H1N1 entre janeiro de 2013 e março de 2014. Sete deles foram transferidos para nosso centro visando à utilização de oxigenação por membrana extracorpórea dentro de um período de 72 horas após o início da ventilação mecânica. A idade mediana foi de 40 anos, sendo 30% dos pacientes do sexo feminino. O valor mediano da proporção entre pressão parcial de oxigênio e fração inspirada de oxigênio foi de 62,5, sendo o escore RESP mediano de 6. Três pacientes receberam inalação de óxido nítrico e quatro utilizaram posição prona como tratamento de resgate antes de ser iniciada a oxigenação por membrana extracorpórea. A duração mediana da ventilação mecânica foi de 22 dias (variação de 14 - 32). O tempo mediano de permanência na unidade de terapia intensiva foi de 27 dias (variação de 14 - 39). O tempo mediano de permanência no hospital foi de 29,1 dias (variação de 16,0 - 46,9). Ocorreram complicações não importantes de sangramento em seis dos dez pacientes. Oito dos dez pacientes sobreviveram até a alta hospitalar.

**Conclusão:**

Os sobreviventes eram relativamente jovens e tiveram alta com boas condições funcionais, o que salienta os anos de vida ajustados pela qualidade que foram salvos. Nossa experiência demonstra que mesmo um programa ainda relativamente novo de oxigenação por membrana extracorpórea pode desempenhar um papel importante, e proporcionar resultados excelentes para os pacientes mais graves.

## INTRODUCTION

Extracorporeal membrane oxygenation (ECMO) has been a therapeutic option for severe acute respiratory distress (ARDS) for approximately forty years.^(1)^ The efficacy of ECMO for treating severe ARDS in adults has been supported by a single randomized controlled trial, which demonstrated a significant improvement in survival, with good neurological function in 180 patients randomized for referral to ECMO consideration *versus* conventional ventilator support (CESAR trial). This study was published in the fall of 2009, just as a global pandemic of a newly emergent H1N1 influenza virus, or swine flu, peaked.

This pandemic originated in Mexico in March 2009,^(2)^ and it was found to have resulted from a quadruple re-assortment between 2 swine, 1 human, and 1 avian influenza strains.^(3,4)^ The epidemiological expression of this pandemic was distinct compared to previous typical seasonal influenza activity.^(5)^ The influenza-related hospitalization rate for adults 18 - 49 years of age increased six-fold.^(6-8)^ Unusually high numbers of young, previously healthy adults suffered severe ARDS requiring mechanical ventilation and, in some centers, ECMO. The Centers for Disease Control and Prevention estimated that 12,500 deaths resulted in the United States,^(6)^ including 85% younger than 65 years of age. When the relative youth of the patients who succumbed was considered, it was estimated that 334,000 - 1,973,000 years of life were lost in the pandemic.^(7)^ The predilection for severe disease in young adults was attributed to the immune naïve status of younger patients in relation to H1N1 influenza strains. Fourteen studies from Europe, Japan, the United States and Australia/New Zealand reported ECMO treatment results from a cumulative population of 487 patients with H1N1 influenza during the 2009 pandemic, with reported survival rates ranging from 32 - 92%.^(9-12)^

The CESAR trial and 2009 flu pandemics were important motivators in the initiation of our ECMO program in May 2010.^(13)^ From 2010-2012, however, H3N2 emerged as the predominant clinical strain of influenza A, and the apparent need for ECMO support for influenza patients at our institution dropped sharply.^(14)^ H1N1 then re-emerged in the fall of 2013.^(15)^ Seasonal influenza A activity in the United States began to increase in mid-November 2013, and referrals of patients with severe ARDS due to influenza A quickly increased at our institution in January 2014. Other ECMO centers in Arizona simultaneously experienced peak ECMO demand. Although we coordinated our efforts, our combined capacity to provide ECMO was nearly completely engaged in February 2014.

This report aimed to describe the outcomes of the patients with severe H1N1 associated acute respiratory distress syndrome who were treated with extracorporeal membrane oxygenation therapy.

## METHODS

The Banner Health Institutional Review Board approved this study, and informed consent and ethical approval requirements were waived (Project # 01-15-0112).

All adult patients who were treated with veno-venous ECMO (vvECMO) for severe microbiologically proven influenza A during the winter of 2013-14 in our medical/surgical intensive care unit at Banner University Medical Center were included. Influenza A virus was detected through polymerase chain reaction, direct fluorescent antigen, rapid enzyme immunoassay test, and/or viral cultures, in clinical specimens obtained through nasopharyngeal swabs, suction of endotracheal secretions or bronchoalveolar lavage. Patients were triaged to vvECMO at the discretion of intensivist and cardiothoracic surgeons. Our triage policy favors vvECMO in patients with an arterial oxygen partial pressure to fraction of inspired oxygen (PaO_2_/FiO_2_) ratio < 100, who do not have life-threatening co-morbidities and who have not received prolonged injurious mechanical ventilation (> 7 days) likely to have resulted in severe ventilator-associated lung injury. Venous access is typically achieved with 27 - 31 F Avalon catheters placed in the right internal jugular vein. The Seldinger technique was used to insert the catheter. Correct positioning was confirmed through fluoroscopy and chest X-ray. Maquet Rotaflow^®^ and Maquet Cardiohelp^®^ ECMO pumps and Quadrox oxygenators were used. The ECMO flow rates were set to achieve arterial oxygen saturations > 85%, while limiting negative inflow pressure to less than 100mmHg. Oxygen was used as our sweep gas and titrated to achieve adequate arterial partial pressure of carbon dioxide (PaCO_2_). Non-adjusted heparin infusions at 1000 units per hour were typically administered. The ventilator settings targeted a lung-rest strategy, generally with fraction of inspired oxygen (FiO_2_) ≤ 50% and peak airway pressures of 20 - 25cmH_2_0.

We collected the following data: the patient demographics, risk factors for severe influenza H1N1 pneumonia and major co-morbidities, respiratory parameters before the ECMO initiation, technical characteristics of ECMO therapy, complications and outcomes. Illness severity was determined using the Sequential Organ Failure Score Assessment (SOFA) and the Acute Physiology and Chronic Health Evaluations score (APACHE IV). Secondary pneumonias were diagnosed through clinical and radiographic findings, in conjunction with quantitative bronchoalveolar lavage cultures.

## RESULTS

A total of 10 patients received vvECMO for H1N1 influenza between January 2013 and March 2014. Seven patients were transferred to our center for ECMO consideration, all within 72 hours of initiation of mechanical ventilation. The clinical features and management, complications and outcomes details are described in table 1. The median patient age was forty years, and 30% were female. Seven patients were obese, with a body mass index greater than 30kg/m^2^. Four patients had significant comorbidities, including chronic obstructive pulmonary disease and coronary artery disease. Most patients, however, were otherwise previously healthy, with few comorbidities. One patient was pregnant and underwent an emergency Caesarean section before starting ECMO. None of our patients had received seasonal influenza vaccinations.

**Table 1:** Baseline characteristics

Patient	Age	Sex	BMI	Co morbidities	Viral testing	Rescue therapy	p/f ratio	SOFA at ECMO initiation	Days on ventilator prior to ECMO
1	27	M	26.5	None	Rapid antigen	Proning	62	13	< 24 hours
2	31	M	41.6	None	Rapid antigen	Prone, Inhaled nitric oxide	44	13	< 24 hours
3	42	M	40	Hypertension, smoking	Viral culture	None	45	10	48 hours
4	44	F	52	Asthma, heart failure	Rapid antigen	Neuromuscular blocking agents, HFOV, inhaled nitric oxide, Prone	43	11	72 hours
5	30	F	NA	Pregnant	DFA	Neuromuscular blocking agents	69	13	24 hours
6	58	M	32	CLL, COPD, smoking	Viral PCR and culture	Bilevel ventilation	66	9	5 days
7	66	M	37	Hypertension, WPW	DFA	Inhaled nitric oxide	56	12	48 hours
8	34	M	33.3	None	PCR	Bilevel ventilation	57	14	24 hours
9	41	F	25.9	Asthma, ulcerative colitis	PCR	None	63	10	24 hours
10	40	M	50	None	PCR	None	49	16	24 hours

BMI - body mass index; SOFA - Simplified Organ Function Assessment; ECMO - extracorporeal membrane oxygenation; M - male; F - female; HFOV - high frequency oscillator ventilation; NA - no assessment; DFA - direct fluorescent antigen testing; CLL - chronic myeloid leukemia; COPD - chronic obstructive pulmonary disorder; WPW - Wolf-Parkinson White; PCR - polymerase chain reaction.

Influenza virus was diagnosed in all patients using at least one of the following viral diagnostic tests: positive reverse-transcriptase polymerase chain reaction (Quest Diagnostics, nine patients), positive viral culture (Quest Diagnostics, two patients), and positive direct fluorescent antibody staining (Quest Diagnostics, three patients). Co-infection with other viruses was found in two patients, one with metapneumovirus and another with respiratory syncytial virus. Nine patients had a bronchoscopy with bronchial wash and lavage performed within 24 hours after hospitalization at our center. Respiratory cultures were sent for viral, bacterial and fungal analysis.

Most patients demonstrated typical symptoms of influenza for at least a week before medical attention was sought but then rapidly deteriorated over 48 - 72 hours. Upon admission, nine patients presented with septic shock requiring vasopressors. The median PaO_2_/Fi0_2_ ratio was 62.5. Six patients had severe and bilateral air space disease involving three or four quadrants. The most common radiological patterns were dense consolidation in all ten patients, ground glass opacities in two patients and pleural effusions in one patient. Interestingly, one patient had a normal chest radiograph upon admission and then, forty-eight hours after admission, progressed to having infiltrates involving four quadrants.

Half of the patients received volume-controlled ventilation, and half received airway pressure release ventilation prior to the initiation of ECMO (Table 2). Oseltamivir was started after a median 24 hours following symptom onset. Three patients received inhaled nitric oxide, and four patients were prone-positioned as rescue therapy before ECMO was initiated. Nine patients were placed on ECMO within 48 hours of admission, and one was placed on ECMO after six days of mechanical ventilation. The median SOFA score at the time of ECMO initiation was 12. Hemorrhagic complications occurred in a total of 6 patients. Four patients suffered bleeding from the ECMO catheter site, which required the temporary discontinuation of heparin. Two patients required transfusions of at least two units of packed red blood cells. Gastrointestinal bleeding was noted in two patients, and it resolved with blood transfusion and temporary withholding of heparin. Entrainment of air into ECMO circuit occurred in one case, but it did not result in systemic air embolization. Six patients suffered seven episodes of secondary bacterial ventilator-associated pneumonia: five due to *Pseudomonas aeruginosa*, one due to methicillin-susceptible *Staphylococcus aureus* and one due to *Enterococcus cloacae*. One patient each suffered acute cardiomyopathy and diffuse alveolar hemorrhage presumably related to influenza. One patient who had underlying lymphoproliferative malignancy developed hemophagocytic lymphohistiocytosis shortly before his death. No patient suffered barotrauma.

**Table 2:** Additional ventilator settings

Patient	Mechanical ventilator settings before ECMO	Mechanical ventilator settings after ECMO	Maximum first day ECMO sweep (L/Min)	Maximum first day ECMO flow (L/min)	Addition of steroids Yes/No	Additional rescue therapies during ECMO
1	APRV, 100 FiO_2_, Th/Tl, 4/0.5s	CMV, TV-500, PEEP-5,50%	2	5	No	None
2	CMV, TV-350, PEEP-16, FiO_2_-80	CMV-350, PEEP-14,50%	6	4.1	No	None
3	CMV, TV- 450, PEEP-20, FiO_2_-80	PS -15, FiO_2_-40	7	4.0	Yes	None
4	HFOV, 100%, Mean pr-35	CMV, TV-200, PEEP-20, FiO_2_-50%	6	4.5	Yes	Proning, inhaled nitric oxide
5	APRV, Th/Tl-3.7/0.3, FiO_2_-100	TV-250, PEEP-20, FiO_2_-50	5	2.7	No	None
6	CMV, TV-350, PEEP-20, FiO_2_-100	APRV, Pr-22/15, Th/Tl-4/0.8, FiO_2_-100	2	4	Yes	None
7	CMV-400, PEEP-20, FiO_2_-100	CMV, TV-350, PEEP-14, FiO_2_-45	4.5	3.3	No	None
8	APRV, Th/Tl-/0.5, FiO_2_-60	APRV-26/16, Th/tl 4/0.5,50%	4.5	3.7	No	None
9	CMV 350, PEEP-15, Fi0_2_-100	CMV, TV-300, PEEP-10, FiO_2_-30	2	3.7	No	None
10	CMV, TV 350, PEEP-20, FiO_2_-100	CMV, TV-350, PEEP-15, FiO_2_-40	7	3.5	No	None

ECMO - extracorporeal membrane oxygenation; APRV - airway pressure release ventilation; FiO_2_ - fraction of inspired oxygen; Th/Tl- time high/time low; CMV - controlled mandatory ventilation; TV - tidal volume; PEEP - positive end expiratory pressure, PS - pressure support; HFOV - high-frequency oscillator ventilation.

The median duration of mechanical ventilation was 22 days (range, 14 - 32), and vvECMO was provided for a median of 12.5 days (range, 8 - 19 days). The median length of stay in the intensive care unit was 27 days (range, 14 - 39), and the median hospital length of stay was 29 days (range, 16 - 46). In two cases, ECMO was terminally withdrawn after the patients failed to recover and after discussions with the surrogate decision makers. Among our eight survivors, all were ambulatory, and seven were on room air at the time of discharge to home.

## DISCUSSION

Here, we describe the outcomes of a cohort of ten patients with severe H1N1 ARDS treated with vvECMO during the winter of 2013-2014 at our institution. The statewide need for ECMO support increased sharply this season compared to prior years, in conjunction with the re-emergence of H1N1 influenza. The observed increase in ECMO utilization was not reflected in the overall mortality of influenza during the 2012-2013 winter, which was not significantly increased compared to the 2010-2012 seasons.^(16)^ It appears that a relatively small subset of young adults who developed severe complications related to H1N1 influenza impacted the ECMO utilization profoundly, without significantly contributing to the much larger overall mortality rate. This phenomenon was also observed in the 2009 pandemic.^(17,18)^

In many aspects, our patients were similar to those reported to have received ECMO for H1N1 influenza in 2009. The median patient age of 40 in our series is similar to that previously reported.^(18-20)^ Other characteristics, such as obesity and comorbidities, were similar to those reported in previous studies.^(9,18,21)^ The SOFA scores of our patients were higher than those reported in a few recently published studies,^(11,18)^ however, the PaO_2_/FiO_2_ ratio and lung injury score were similar to those in other reported studies.^(10,17,18)^ ECMO was started within 48 hours of mechanical ventilation in nine patients, similar to other studies.^(11,17,18,21)^

Life-threatening complications were common in our cohort (Table 3). The most frequent complication seen in patients treated with ECMO was bleeding.^(22-27)^ Cannula insertion sites, which were the most frequent bleeding site reported in the Extracorporeal Life Support Organization (ELSO) registry (occurring in 17% of patients), were also the most frequent bleeding site in our study.^(26)^ Nine patients suffered septic shock, and six required continuous renal replacement therapy. Our patients suffered a high rate of ventilator-associated pneumonia (VAP), despite active quality improvement efforts at our hospital to prevent VAP. The incidences of secondary bacterial VAP have ranged from 40 to 71% in previous studies of influenza patients on ECMO,^(9,12,23)^ and we are not the first group to report a preponderance of gram-negative pathogens.^(9,28)^ It is likely that post-influenza bacterial pneumonias are fundamentally different than VAP in non-influenza patients and more difficult to prevent. Less common complications, such as diffuse alveolar hemorrhage, myocarditis and hemophagocytic lymphohistiocytosis, significantly impacted morbidity and mortality rates in our patients. Virus-associated hemophagocytic syndrome has been reported to be a major cause of death in 9 of 17 patients who received ECMO for H1N1 Influenza related ARDS.^(29)^ Renal dysfunction is also a common complication, with six patients requiring continuous renal replacement therapy, and it reportedly occurred in 13% of patients in the ELSO registry.^(26)^ In an analysis of 72 patients receiving ECMO for respiratory failure, only pre-ECMO serum creatinine levels correlated with survival.^(30)^ It is unclear if this finding is related to the overall severity of organ dysfunction and not specifically to ECMO.

**Table 3:** Complications

Complications	N
Septic shock requiring vasopressors	9
Acute renal failure requiring continuous renal replacement therapy	6
Viral cardiomyopathy	1
Diffuse alveolar hemorrhage	1
Ventilator associated pneumonia	6
Hemophagocytic lymphohistiocytosis	1

Despite the complications described above, our outcomes were encouraging and comparable to those reported during the 2009 H1N1 pandemic (Table 4). Our 80% survival rate is similar to that reported by Australia and New Zealand Extracorporeal Membrane Oxygenation (ANZ ECMO) Influenza Investigators (79%), Zangrillo et al. (72%), Pham et al. (64%) and Holzgraefe et al. (92%).^(9,11,18,21)^ A small study in a French hospital (n = 12) reported a high survival rate, despite many complications, such as VAP, which was observed in 6/12 patients, and major hemorrhage, which was reported in 8/12 patients.^(25)^ Our surviving patients were all discharged with good functional status. Other small observational studies have reported lower survival rates, including a 35% survival rate reported in a Japanese series (n = 14), 39% survival in a series from Germany (n =18), 44.4% in some French centers (n = 9) and a 44.4% survival rate reported by Spanish hospitals (n = 9).^(19,22,24,27)^ While these findings could be attributed to hemorrhagic complications and longer durations to the initiation of ECMO, lack of experience and technical challenges could also have influenced survival rates.^(19,22,24)^ Although ECMO is an expensive support modality, its cost effectiveness is enhanced in the treatment of relatively young patients likely to enjoy good functional status at discharge. Such patients are likely to enjoy many future years of high-quality life.

**Table 4:** Outcomes

Outcomes	N
Median duration of days on ECMO	12.5 (8 - 19)
Median duration of days on mechanical ventilation	22 (14 - 32)
Median duration of days in the intensive care unit	27.5 (14 - 39)
Survivors	8 (80%)

ECMO - extracorporeal membrane oxygenation.

Regional ECMO triage was an important factor related to our patient series. It was our shared statewide experience that influenza A caused many more cases of severe ARDS requiring vvECMO in 2013-2014 compared to any previous recent year, with the possible exception of 2009. To the best of our knowledge, this result was a legitimate reflection of the virulence of the strain and not due to triage bias. By January 2014, our ECMO utilization was the highest that we had experienced in the short history of our program. At several points over the winter, all four of our available ECMO units were in use, necessitating the loan of a backup circuit. A statewide organization of ECMO providers was formed to discuss management and provide a state-wide triage system in which patients could be transferred from one ECMO center to another (if needed), depending on the availability of ECMO circuits/pumps. This system was effective in placing all patients for whom ECMO was indicated in a center (located somewhere in the state) that could provide the required care. We believe that this cooperation was instrumental in the high survival rate reported here.

## CONCLUSION

H1N1 might re-emerge in the future and cause severe respiratory disease, despite the increasing immunity of our population. Other respiratory viruses, such as H5N1 avian influenza and Middle Eastern respiratory virus MERS, may also emerge to burden regional extracorporeal membrane oxygenation capacities with unpredictable timing. We believe that extracorporeal membrane oxygenation capacity should be regionally planned and that triage should be regionally coordinated. Our experience shows that even a relatively new extracorporeal membrane oxygenation program can play an important role in that capacity and provide excellent outcomes for the sickest patients.
